# Auxiliary midwives in hard to reach rural areas of Myanmar: filling MCH gaps

**DOI:** 10.1186/s12889-016-3584-x

**Published:** 2016-09-01

**Authors:** Sangay Wangmo, Rapeepong Suphanchaimat, Wai Mar Mar Htun, Tin Tun Aung, Chiraporn Khitdee, Walaiporn Patcharanarumol, Pe Thet Htoon, Viroj Tangcharoensathien

**Affiliations:** 1 International Health Policy Program (IHPP), Ministry of Public Health, Tiwanon Road, Nonthaburi 11000 Thailand; 2 MoH, Myanmar, Naypyitaw, Myanmar; 3 Independent consultant, Naypyitaw, Myanmar

**Keywords:** Auxiliary midwife, Frontline health volunteers, Human resource for health, Maternal and child health, Task Shifting, Health system strengthening, Myanmar

## Abstract

**Background:**

Auxiliary Midwives (AMWs) are community health volunteers supporting the work of midwives, especially maternal and child health services in hard to-reach areas in Myanmar. This paper assessed the contributions of AMW to maternal and child health services, factors influencing their productivity and their willingness to serve the community.

**Method:**

The study applied quantitative cross-sectional survey using census method. Total of 1,185 AMWs belonging to three batches: trained prior to 2000, between 2000 and 2011, and in 2012, from 21 townships of 17 states and regions in Myanmar participated in the study. Multiple logit regression was used to examine the impact of age, marital status, education, domicile, recruitment pattern and ‘batch of training’, on AMW’s confidence level in providing care, and their intention to serve the community more than 5 years.

**Results:**

All AMWs were able to provide essential maternal and child health services including antenatal care, normal delivery and post-natal care. They could identify and refer high-risk pregnancies to larger health facilities for proper management. On average, 9 deliveries, 11 antenatal and 9 postnatal cases were performed by an AMW during the six months prior to this study. AMWs had a comparative advantage for longer service in hard-to-reach villages where they lived, spoke the same dialect as the locals, understood the socio-cultural dimensions, and were well accepted by the community. Despite these contributions, 90 % of the respondents expressed receiving no adequate supervision, refresher training, replenishment of the AMW kits and transportation cost. AMWs in the elder age group are significantly more confident in taking care of the patients than those in the younger groups. Over 90 % of the respondents intended to stay more than five years in the community. The confidence in catering services appeared to have significant association with a longer period of stay in AMW jobs as evidenced by the odds ratio of 3.5, compared to those reporting unconfident.

**Conclusions:**

Comprehensive support system and national policy are needed to sustain and strengthen the contributions of AMWs, in sharing the workload of midwives, particularly in hard-to-reach areas of Myanmar.

**Electronic supplementary material:**

The online version of this article (doi:10.1186/s12889-016-3584-x) contains supplementary material, which is available to authorized users.

## Background

The Republic of the Union of Myanmar is in the middle of vibrant political, social and economic transitions. Despite the recent positive economic performance, Myanmar is classified as lower middle income with estimated GNI per capita of $1280 in 2015 [1]. Myanmar was still not on track in achieving MDG by 2015, especially its infant and child mortality indicators [2]. On the progressive note, health expenditure in Myanmar had increased from 1 % of total government expenditure in 2005 to 3 % in 2012 [3], although at a very low level, it demonstrated strong political commitment to health.

Severe shortage of human resources for health (HRH), impedes the performance of many countries in achievement of Millennium Development Goals (MDGs) 4 and 5 [4, 5]. Further the challenge of deployment, retention, distribution and performance of health workforce, experienced by countries of all socio economic conditions, makes it worse [6–9]. Myanmar for one is among 57 countries experiencing critical shortage of health workforce with 1.61 doctors, nurses and midwives per 1,000 population [10]. This falls below the suggested threshold of 2.28 per 1,000 population, a level that ensures high coverage of essential health services [11]. Not only is there an inadequacy of health workforce but also the maldistribution is jeopardizing access to services, resulting in poor health status amongst people living in hard-to-reach areas. Internal migration of health workers from rural to urban areas, from the public to the private sector and from primary health care level to the higher-up care level and some quitting from the profession, all exacerbate the health workforce scarcity and jeopardize the achievement of health related MDG.

Public health in Myanmar faced critical resource constraints, creating major gaps in access to, and coverage of health services [12]. Myanmar’s slow progress on key maternal health indicators, including skilled birth attendance, antenatal care coverage between 1990 and 2010 [13, 14], has strong linkage to human resource shortage, particularly in the hard to reach and remote areas. Country’s current health workforce strategic plan recommends continued commitment from government to invest in training and to ensure rural retention of health workforce [15].

Varieties of community-based practitioners are used in escalating access to essential health services, mainly in the under-served communities of low- and middle- income countries [16–18]. Among them, auxiliary health workers are increasingly becoming the main providers of health services in many countries. For instance, despite the professional restrictions and regulations, auxiliary workers like nurse aids and clinical officers in Tanzania and Uganda provides essential medical tasks, in rural areas [19]. In many countries, auxiliary nurses and lay health workers are well accepted and recognised by the community, for their contribution in enhancing availability and accessibility of health services at lower costs, and in addressing the shortage or misdistribution of human resources for health [20].

Recognising its benefit, Myanmar also trained and deployed Auxiliary Midwives (AMWs) to perform as extension arms of limited number of trained health workforce especially midwives, in delivering MCH services to the hard-to-reach and remote areas. The Ministry of Health (MOH) executes the AMW training programme by mobilising funding from various sources, either from the MOH’s core budget or international development partners when opportunities arose.

In addition to introducing new and under-used vaccines, the Global Alliance on Vaccine and Immunization (GAVI) had provided Myanmar with funding to support Health Systems Strengthening (HSS) programme. The GAVI HSS supported recruitment and training of new AMWs, as well as refresher courses for the existing AMWs in the townships implementing GAVI HSS. Due to resource constraints, each township had annual quota of 20 seats for new recruitment of a six-month training to be an AMW, and an annual quota of 50 seats for AMWs trained after 2012, to attend refresher course.

As a part of routine monitoring and evaluation of GAVI HSS project in Myanmar, a quantitative cross-sectional study was conducted in 2013, to assess the extent of AMW’s contribution in addressing the shortage of midwives, by assisting the existing midwives in delivering MCH services to the communities of hard to reach areas. This study aimed to understand AMW’s strengths, weakness and support needed, to better inform future policy decision in strengthening the AMW program in Myanmar.

## Methods

### Study design

This study applied cross-sectional design, using quantitative self-administered questionnaire. The study was carried out in nineteen townships where the GAVI HSS program was implemented, plus two new townships (Pinlong and Thanatpin) where the GAVI HSS was about to commence by 2014.

As the study aimed to assess all AMWs in the programme, a census was applied instead of a random sampling survey. Questionnaires were distributed to Basic Health Staff (BHS) of all health facilities in these 21 townships, at the July 2013 township monthly meeting. The BHS circulated the questionnaires to all AMWs in their catchment area after the meeting. After reading and signing the consent form on the first page of questionnaire, respondents were requested to answer the questionnaires independently; BHS then collected the completed questionnaires. Since it was difficult to gather all AMWs in one place, some BHS outreached their houses and collected the filled questionnaires.

### Pilot test and questionnaire distribution

The first draft questionnaire was tested on the relevance of contents (content validity) to AMW in the catchment areas of two selected rural health centres in two townships, one as best and the other worst performing, in view of the township medical officer. The questionnaire was already translated to Burmese language prior to the test. A total of 33 AMWs participated in the test.

After piloting, contents of the questionnaire were reviewed, revised and finalized before submission to the selected townships. The completed questionnaires were collected from BHS during the township monthly meeting in August 2013. All questionnaires were sent to the WHO country office in Yangon by pouch.

### Questionnaire design

The contents of questionnaire were divided into three parts. The first part included questions on personal attributes, such as age, marital status, education, domicile (if they were living in the village they served, or otherwise) and main job. The second part covered history of being an AMW, who proposed them for training, their main motivation, when were they newly trained or refreshed (recent GAVI batch in 2012, older batches between 2000 and 2011 and oldest batch prior to 2000), and their level of confidence to provide service to villagers (Likert –type scale from 1 to 4, from very confident to not confident). The third part was related to their contributions to villages, including number of high risk pregnancies identified and referred, and number of refusal to referral, number of antenatal care, delivery and postnatal care they had provided in the last six months, their needs for supports, such as incentives for their work, transport, and or refresher training, their perception of being accepted by the communities, support they got from communities and patients they served, and satisfaction with supports from rural health centre (RHC) and sub-centre. This section ended up with number of years they intended to serve the communities and reasons to quit from AMW (arranged in Likert-typed scale: 1 as ‘less than one year’, 2 as ‘1–3 years’, 3 ‘3–5 years’, and ‘ > 5 years’.

### Data analysis

Data were entered in Excel and were transferred to STATA XI (STATA ® 12, Stata Corporation, College Station, Texas, USA) for the analysis. The analysis was composed of two steps. Firstly, the descriptive statistics using means and frequency were applied to explore general information of the respondents’ characteristics. Secondly, inferential statistics, including multiple logit regression, were exercised.

The dependent variables for the analysis in the second step were (1) confidence level in providing care, and (2) intention to serve the community more than 5 years. These variables were converted from ordinal scale variables to binary variables. That is, respondents answering 1 (very confident) or 2 (confident) in the question on level of confidence were coded as 1 (confident) and 0 (not confident) if otherwise. Those, who reported intending to continue their stay in the community for more than 5 years (answering choice 4 in the question), were coded as 1 (intending to stay) and 0 (not intending if otherwise).

In the multiple logit regression, these dependent variables were regressed one by one on all of the following independent variables, namely, ‘age group’, ‘education level’ (grade 5, 9, 11 and bachelor or above), ‘recruitment pattern’ (self-application, proposed by midwives, villagers and village head), and ‘batch of training’ (before 2000, 2000–2011 and 2012) included in the model.

Notice is made that in the inferential analysis of intention to serve the community beyond the next 5 years, an additional independent variable, that is level of confidence (which served as the dependent variable in the previous model) was put into the model as well, since the researchers hypothesised that the confident workers were more likely to show interest in engaging in AMW job for a longer period of time than the unconfident ones.

The decision of selecting variables into the model was based on consultative meetings between the research team and external experts held in WHO Country Office Myanmar. The variables selection was derived from prior knowledge in academic literature [21] that, aside from the programme determinants, such as training batch and how they were recruited to the programme, background of the respondents (such as age and education level) is of importance in determining the confidence in providing care and intention to serve the community. P-value of 0.05 was used as a cut-off point for statistical significance.

## Results

1. Profiles of AMW
  From the census, total 1,185 AMW from 21 townships completed the questionnaire; all were women and about half were married. The majority of respondents were in an age group of 30 years or more, and completed their education up to grade 11. This reflected that most AMW were educated at least up to essential education, though very few of them finished a degree level.
  Almost all of the respondents (98 %) lived in the village where they provided services. Being AMW was a voluntary job as all respondents already had other main occupations; about three quarters of them earned living from farming, see Table 1.

**Table 1:** Profiles of auxiliary midwives (*N* = 1,185)

Characteristics	Frequency	Percent
Age		
< 20 years	83	7.1
20–29 years	397	33.9
30–39 years	312	26.6
40–49 years	191	16.3
> = 50 years	188	16.1
Education		
Up to grade 5	151	12.8
Up to grade 9	403	34.3
UP to grade 11	554	47.1
Degree or above	68	5.8
Domicile		
Living in the service area	1,139	97.6
Living outside the service area	28	2.4
Main occupation		
Farming	800	71.1
Shop owner	192	17.1
Others	133	11.8

Note: Missing data are not included in the analysis
2. Becoming an auxiliary midwife: the pathways
  The majority of AMW (59 %) came to this work from self-application. The most important reason for joining this programme was the chance to serve the community. For reasons of becoming AMW, altruism was the main motivation to be an AMW, as 89 reported this was a chance to serve people in their own village, and 6 % stated that they wished to be upgraded to a midwife. Less than half percent reported a chance to earn some money from their services. About 39 % of the respondents were trained before 2000 and over half of the respondents ever attended refresher course.
  After the six-month training course, over four fifths of the participants reported ‘fairly confident’ to ‘very confident’ in providing care, see Table 2.

**Table 2:** Factors and pathway to become auxiliary midwives

Variables	Frequency	Percent
Proposing person		
Village head	172	15.0
Villagers	76	6.6
Midwives	228	19.9
Self applying	671	58.5
Reasons for application		
Having chance to serve villagers	1,002	88.5
Being recognised by community	64	5.6
Having chance to be upgraded to midwife	63	5.6
Earning more revenue	3	0.3
Batch		
Year 2012	308	26.7
Year 2000–2011	396	34.4
Before 2000	449	38.9
Attending refresher course (in 2012)		
Ever attended	513	58.2
Not attended	368	41.8
Confidence after attending refresher course		
Very confident	289	25.0
Confident	693	60.1
Fairly confident	162	14.0
Not confident	7	0.6
Not confident at all	3	0.3
3. AMW: contributions of and supports to AMW
  In the past six months prior to the survey, on average, 2.9 high-risk pregnancies were identified and referred by them to the respective Township Hospital. The participants also reported they had assisted an average of nine deliveries in the last six months, eleven antenatal care cases and nine postnatal cases. Concerning support from the sub-centre or RHC, only 3 % of respondents answered ‘not satisfied’ to the support received. The survey found that 72 % of them reported they were well accepted by their community, and only infinitesimal group of respondents reported not being accepted by the villagers.
  Approximately two thirds of the respondents reported difficulty in travelling to households of women and children in need in remote or hard-to-reach areas. Concerning sources of financial assistance, about 9 of the respondents received financial support from the community and 39 % got payments from the patients they served.
  The majority of respondents opined that they still needed support in the following items for improving their quality of care, such as technical supervision (99), refresher course (99), replenishment of AMW kits (96), and transportation to reach hard-to-reach villagers (74 %), see Table 3.

**Table 3:** Contribution of AMW and their needed support

Contributions of AMW	Mean	Standard error
Number of referred pregnant cases	2.9	5.2
Number of deliveries	9.0	29.2
Number of cases received antenatal care	11.5	29.9
Number of deliveries in the last six month	9.2	34.4
Support to AMW	Frequency	Percent
Support from rural health centre		
Very satisfied	325	28.0
Satisfied	674	58.1
Fairly satisfied	112	9.7
Unsatisfied	38	3.3
Very unsatisfied	10	0.9
Travelling difficulty to hard to reach areas		
Having difficulty	692	65.5
Not having difficulty	395	34.5
Experiencing patients refusing to be referred to		
higher facilities		
Ever experienced	546	57.2
Never experienced	408	42.8
Sources of financial assistance		
From community fund		
Ever received	89	8.7
Never received	932	91.3
From individual patients		
Ever received	414	39.0
Never received	649	61.0
Needed support		
Technical supervision		
Needed	1100	99.1
Not needed	10	0.9
Refresher course		
Needed	1054	99.0
Not needed	11	1.0
Kits replenishment		
Needed	949	96.0
Not needed	40	4.0
Transportation support		
Needed	691	74.0
Not needed	243	26.0
4. AMW: Intention to stay
  A futuristic question was asked on how many years they intended to serve as AMW. The majority, 90 %, reported an intention to serve the community more than 5 years, whilst only 1 % expected to be engaged in this work for less than a year. The main reasons of quitting were; if they needed to change their domicile (21) and if they were not able to contribute to the community as intended (23 %), see Table 4.

**Table 4:** Expected years to serve the community and potential reasons for quitting AMW job

Variables	Frequency	Percent
Expected years to serve the community		
< 1 years	12	1.1
1–3 years	58	5.1
3–5 years	39	3.5
> 5 years	1019	90.3
Reasons for quitting AMW jobs		
Marriage		
May consider quitting jobs	45	4.2
Not relevant to the decision of quitting jobs	1023	95.8
Changing domicile		
May consider quitting jobs		
Not relevant to the decision of quitting jobs	218	21.3
Having another permanent job	806	78.7
May consider quitting jobs		
Not relevant to the decision of quitting jobs	124	12.4
Cannot contribute to the community as much as expected	878	87.6
May consider quitting jobs	228	22.6
Not relevant to the decision of quitting jobs	779	77.4
5. AMW: confidence and intention to contribute more than five years
  Multiple logit regression reveals that the older AMW were, the more confident in providing service. Those aged 50 or more tended to be approximately 5 folds more confident than the youngest age group with statistical significance. Conversely, AMW who completed up to grade five, those who self-applied, and were trained before 2000, were likely to report having confidence in service provision despite without statistical significance, see Table 5.

**Table 5:** Multiple logit regression: factor contributing to confidence in providing services

Feeling confident	Odd ratio	*P*-value	95 % CI	
Age (versus < 20 years)				
20–29	1.038	0.899	0.583	1.850
30–39	1.493	0.257	0.747	2.981
40–49	6.019	0.000	2.259	16.038
> = 50	4.966	0.001	1.892	13.031
Education (versus up to grade 5)				
Up to grade 9	0.713	0.347	0.353	1.443
Up to grade 11	0.642	0.214	0.319	1.291
Up to bachelor or above	0.478	0.111	0.192	1.186
Recruitment by (versus self applying)				
Local midwives	0.944	0.788	0.623	1.433
Villagers	0.655	0.216	0.335	1.280
village head	0.866	0.581	0.520	1.444
Training batch (versus year 2000)				
2000–2011	1.703	0.062	0.974	2.977
2012	1.062	0.849	0.574	1.963
  Results from multiple logit regression, where intention to contribute more than 5 years serving as the dependent variable is demonstrated in Table 6. It is clear that the confident respondents showed higher probability to stay longer than 5 years as presented by the odds ratio of 3.5 with statistical significance. Also the older the respondents were, the higher was the probability of intending to stay more than five years but without statistical significance.

**Table 6:** Multiple logit regression: factor contributing to intention to contribute more than five years

Intention to serve communities >5 years	Odd Ratio	*P*value	95 % CI	
Confidence in providing services (VS not confident)	3.486	<0.001	2.195	5.537
Age (versus < 20 years)				
20–29	1.327	0.382	0.703	2.506
30–39	1.783	0.171	0.779	4.079
40–49	1.710	0.330	0.582	5.025
> = 50	2.532	0.147	0.720	8.901
Education (versus up to grade 5)				
Up to grade 9	0.345	0.096	0.099	1.207
Up to grade 11	0.255	0.031	0.074	0.881
Up to bachelor or above	0.531	0.441	0.106	2.657
Recruitment by (versus self applying)				
Local midwives	0.726	0.247	0.422	1.248
Villagers	0.324	0.004	0.150	0.701
village head	0.300	<0.001	0.168	0.535
Training batch (versus year 2000)				
2000–2011	0.655	0.301	0.294	1.460
2012	0.252	0.001	0.109	0.582
  The respondents with an education background more than grade 5 were likely to engage in AMW work for a shorter period of time than those completing up to grade 5. Those recruited to the training programme through other means tended to contribute to this work shorter than the self-applicants. This phenomenon was observed in those trained in the later batches, particularly the participants in the 2012 batch was about 75 % less likely to serve the community for more than 5 years, compared to those received training before 2000 with statistical significance.

## Discussion

According to our study, AMW has comparative advantage to be effective community health workforce, as they live in the hard-to-reach villages, speak the same dialect as the locals, understand the socio-cultural dimensions, and are well accepted by the community. This was also confirmed by a UNICEF study:

*….because of easy access, close social relationships, personal trust and social convenience, use of AMW was very high especially in remote villages with limited geographical accessibilities. The same was true for AMW residing in villages far from other health care facilities. Moreover, AMW were utilized in villages as a substitute for Traditional Birth Attendants, who became defunct due to senility or disabilities* [22].

Auxiliary health workforce or community health volunteers though seen as a good strategy to fill the human resource gaps, particularly in the remote and rural areas, difficulty to retain and maximize the contributions by these community health volunteers is a challenge faced by many countries [23]. Exploring for solution, studies indicated work environment [24], altruism, social recognition, knowledge gain and career development as different motivation factors for lay health workers (LHW) [25]. Similarly, altruism was the main motivation to be an AMW in Myanmar. Further, financial incentive, medical supplies, and timely replenishment of AMW kits with adequate supervision, are critically needed to retain and improve the productivity of AMWs.

Sustainable and standardised financial incentive is needed not only to motivate the AMWs, but also to avoid financial burden on the rural community. For instance, AMWs make substantial investment for their travel costs when visiting people’s homes, time costs when they must get time-off from their routine productive work, and for the purchase of medicines and other basic medical supplies. Unavoidably, these costs are shouldered either by the community or the patients. Naturally in Myanmar, 39 of AMWs received financial support from the patients they served and 9 % from the community, which can create financial burden on the rural community.

AMW kits are critical tools, necessary in performing their core functions of ensuring sanitary and safe conditions for woman in labour, during delivery and postnatal care. The kits contain a plastic sheet, a bar of soap, a two-sided clean razor blade, cord ties, and pictorial instructions on how to deliver a child. All these are consumables and need a system of regular replenishment to avoid service disruption.

While recognising the value of community health volunteers, evidence to prove their contribution is essential to sustain policy support [17]. Findings from our study indicated their meaningful contribution in delivering MCH services. It showed, in the last six months prior to the study, AMW assisted an average of nine deliveries, eleven antenatal care cases and nine postnatal cases, which was similar to UNICEF study--an average 10.3 assisted delivery [22]. AMW’s contribution in delivering MCH services is further confirmed through another study in Kyimyindaing Township in Myanmar. According to that particular study, urban women in Kyimyindaing Township received ANC mainly from specialist and medical doctors (68.7 %); yet majority of rural women consulted AMWs (84 %) for ANC services [26]. Hence, essential it is to enhance the support system to strengthen and sustain their contribution.

Assessing the determinants of attrition of auxiliary health workers is critical for their retention, as not many studies focused on this [27]. Responding to such need, our study explored the relationship between age, education and recruitment pattern of AMW, and their willingness to continue service. In Myanmar, age group and recruitment pattern of AMWs play a key role in their attrition. The older the AMW, the higher is the probability of their intention to serve more than five years.

Further, AMWs who volunteered their application, and those who applied prior to year 2000 had higher confidence to serve more than 5 years. Such findings clearly indicate the need for policy intervention and system support to provide regular refresher courses for the older and senior volunteers, to ensure service quality. On the other hand, it also questions the scaling up potential and sustainability of AMW program in the longer run, as the newer batch of AMWs are less keen to continue service. Hence, comprehensive streamlining of AMW program in Myanmar is worthwhile, to attract more new volunteers to sign up, and motivate all existing ones to continue, and provide quality services.

Responding to shortage of health workforce, WHO recommends to train and empower “mid-level’ and ‘lay’ health workers to perform specific interventions that may else be performed by specialized cadre of health workers, to improve access to care [28]. Consequently, task shifting defined as the process of delegation, where tasks are moved to less specialized health workers [29], was commonly practiced in several countries facing critical shortage of health workforce, notably in South Asian and Sub-Saharan African countries.

Training and deployment of AMW is clearly supporting this recommendation by shifting some of midwives task to them especially in hard-to-reach areas. A successful task shifting requires a comprehensive national policy and guidelines where legal protection is in place for those undertaking additional tasks, clear policies toward supporting them to provide quality service, under close supervision and support.

Despite the merits of task shifting as an important policy option to improve workforce shortages; there are several concerns, for example, task shifting in HIV/AIDS in sub-Saharan Africa noted quality and safety concerns [30], professional and institutional resistance, and the need to sustain motivation and performance [31].

The limitation of this study is lack of assessment of the quality of services provided by the AMWs. Further, due to time and data limitation, it could not assess the proportion of contributions made by AMWs, as compared to other categories of health workers, particularly the midwives. Besides, the questionnaire is self-reported and this made it difficult to control the quality and honesty of reporting.

## Conclusion

Comprehensive policy interventions and adequate support systems are critical to sustain and strengthen the contributions of AMWs in sharing the workload of BHS. Health volunteers, such as AMWs are recognised as extremely important in supporting the achievement of Health related MDGs and SDGs in many low-income countries [32, 33].

In addition to strengthening the AMWs, Myanmar must adopt the long-term solution of training more midwives and retain them in rural hard to reach areas, as recommended by WHO [34], such as recruitment of young rural women for midwifery training, strengthen midwifery training institutes. Also intensify policy interventions to ensure a high level of retention in rural areas, through hometown work placements upon graduation, financial and non-financial incentives; provision of adequate medicines and supplies, support and supervision by township peers, and daily travel allowances.

## Additional file

Additional file 1: Questionnaire. (PDF 125 kb)

## Abbreviations

AIDS: Acquired immune deficiency syndrome
AMW: Auxiliary midwife
BHS: Basic health staff
GAVI: Global alliance on vaccine and immunization
GNI: Gross national income
HIV: Human immunodeficiency virus
HRH: Human resource for health
HSS: Health system strengthening
LHW: Lay health workers
MDG: Millennium development goal
MOH: Ministry of health
ODA: Official development assistance
RHC: Rural health centres
UNICEF: United Nations international children’s emergency fund
WHO: World Health Organization
