# Erratum to: Auxiliary midwives in hard to reach rural areas of Myanmar: filling MCH gaps

**DOI:** 10.1186/s12889-016-3747-9

**Published:** 2016-10-12

**Authors:** Sangay Wangmo, Rapeepong Suphanchaimat, Wai Mar Mar Htun, Tin Tun Aung, Chiraporn Khitdee, Walaiporn Patcharanarumol, Pe Thet Htoon, Viroj Tangcharoensathien

**Affiliations:** 1 International Health Policy Program (IHPP), Ministry of Public Health, Tiwanon Road, Nonthaburi, 11000 Thailand; 2 MoH, Myanmar, Naypyitaw, Myanmar; 3 Independent consultant, Naypyitaw, Myanmar

## Erratum

In the publication of this article [1], the following percentage symbols were accidentally missed out from the ‘Results’ and ‘Discussion’ sections:

**Results**

2. Becoming an auxiliary midwife: the pathways

…as 89 % reported…

3. AMW: contributions of and supports to AMW

…about 9 % of the respondents…

…such as technical supervision (99 %), refresher course (99 %), replenishment of AMW kits (96 %)…

4. AMW: Intention to stay

…if they needed to change their domicile (21 %)

**Discussion**

…Naturally in Myanmar, 39 % of AMWs…

In addition to the above, some figures in Table 4 were misaligned. The updated Table 4 is presented below:

**Table 4:** Expected years to serve the community and potential reasons for quitting AMW job

Variables	Frequency	Percent
Expected years to serve the community		
< 1 years	12	1.1
1–3 years	58	5.1
3–5 years	39	3.5
> 5 years	1019	90.3
Reasons for quitting AMW jobs		
Marriage		
May consider quitting jobs	45	4.2
Not relevant to the decision of quitting jobs	1023	95.8
Changing domicile		
May consider quitting jobs	218	21.3
Not relevant to the decision of quitting jobs	806	78.7
Having another permanent job		
May consider quitting jobs	124	12.4
Not relevant to the decision of quitting jobs	878	87.6
Cannot contribute to the community as much as expected		
May consider quitting jobs	228	22.6
Not relevant to the decision of quitting jobs	779	77.4

All the above have been updated in the original article.
