# Supplementary material for: Auxiliary midwives in hard to reach rural areas of Myanmar: filling MCH gaps
Source: BMC Public Health. 2016 Sep 1;16(1):914. doi: 10.1186/s12889-016-3584-x (PMC5007995; doi:10.1186/s12889-016-3584-x)
Supplement: Additional file 1: — Questionnaire. (PDF 125 kb) [file 12889_2016_3584_MOESM1_ESM.pdf]

Township Code ☐☐☐

Auxiliary Midwives: the contributions of health volunteers in hard-to-reach areas in Myanmar

Objectives

- To assess the socio-economic profile of auxiliary midwives (AMW), their contributions to fill the gap of MCH service in hard to reached areas and needs for supports for the improvement of the program.
- We will keep your information strictly confidential

Informed consent

Having read the objectives, I agree to participate with this research.

I. Your personal background

1. Your age range? please ☒ only one
   ☐ 1 < 20 ☐ 2 20-29 ☐ 3 30-39 ☐ 4 40-49 ☐ 5 50+
2. Your marital status? please ☒ only one
   ☐ 1 Single ☐ 2 Married ☐ 3 Other
3. Your highest education level? please ☒ only one
   ☐ 1 Grade 5 ☐ 2 Grade 9 ☐ 3 Grade 11 ☐ 4 Bachelor degree
4. Do you currently live in the village where you served? please ☒ only one
   ☐ 1 Yes ☐ 2 No
5. Do you speak the same dialect with people live in the village you served? please ☒ only one
   ☐ 1 Yes ☐ 2 No
6. What is your current main job, in addition to your voluntary work as auxiliary midwife,? please ☒ only one
   ☐ 1 Farming, agriculture
   ☐ 2 Shop keeper
   ☐ 3 Others such as teacher, teacher assistant, baby sitter, etc

II. About being a auxiliary midwife

7. Who proposed you to be trained as an Auxiliary Midwife? please ☒ only one
   ☐ 1 Village head
   ☐ 2 Villagers
   ☐ 3 A midwife or a staff at a sub-centre or a rural health centre
   ☐ 4 I applied myself

Township Code ☐☐☐

8. What is your main motivation of being an Auxiliary Midwife? please ☒ only one

☐1 I am proud of myself of having a chance to serve people

☐2 I will be recognized by the community for my contribution

☐3 I may have a chance to upgrade training to be a midwife

☐4 I may earn some income from these voluntary services

9. When were you trained in 6 months course to be an AMW? please ☒ only one

☐1 2012

☐2 Between 2000 – 2011

☐3 Before 2000

10. Did you get 5 days refresher course? please ☒ only one applicable to you

- in 2012 ☐1 Yes ☐2 No
- between 2000-2011 ☐1 Yes ☐2 No
- before 2000 ☐1 Yes ☐2 No

11. After the training, how confident you are in providing services to the villagers? please ☒ only one

☐1 Very confident ☐2 Confident

☐3 Fairly confident ☐4 Not confident ☐5 Not confident at all

III. About your contributions to villagers

12. After the training, did you satisfied with supports you receive from the sub-centre or the rural health centre? Please ☒ only one

☐1 Very satisfied

☐2 Satisfied

☐3 Fairly satisfied

☐4 Unsatisfied

☐5 Very unsatisfied

13. You contributions after the training in the past 6 months,

- Number of high risk pregnancies you had referred? ☐☐ Cases
- Number of high risk pregnancies who refused referral? ☐☐ Cases

Some key services you provided in the last 6 months

- Delivery ☐☐☐ Cases
- Antenatal care ☐☐☐ Cases
- Post natal care ☐☐☐ Cases

Township Code

14. Do you think the lists below are important to improve your work or not?

- Technical supervision by Midwife and other health staffs ❑1 Yes ❑2 No
- Replenishment of AMW Kit ❑1 Yes ❑2 No
- Financial support for work ❑1 Yes ❑2 No
- Financial support for transport ❑1 Yes ❑2 No
- Refresher Training Course ❑1 Yes ❑2 No

15. Do you think the community accepts the works you contributed or not?? Please ☒ only one

❑1 Yes, very well accept

❑2 Somewhat accept

❑3 No, not accept

16. Do you think the lists below make you difficult to provide services to villages or not?

,

- ❑1 Difficulty to travel and reach women and children ❑1 Yes ❑2 No
- ❑2 Patient refusal to be referred ❑1 Yes ❑2 No
- ❑3 Low educated and lack of health literacy among women ❑1 Yes ❑2 No
- ❑4 Lack of awareness and interests by villagers for health improvement ❑1 Yes ❑2 No
- ❑5 Lack of support from basic health staffs ❑1 Yes ❑2 No

17. Do you get financial support in return of your works?

- From the community ❑1 Yes ❑2 No
- From the patients ❑1 Yes ❑2 No

18. How many years you intended to serve as an auxiliary midwife? please ☒ only one

❑ Less than 1 year ❑ 1-3 years ❑ 3-5 years ❑ > 5 years

If you will quit from Auxiliary Midwife, what are the reasons?

- I may get married ❑1 Yes ❑2 No
- I may move out from the village ❑1 Yes ❑2 No
- I may get a permanent job or employment ❑1 Yes ❑2 No
- I may not contribute as much as I expect ❑1 Yes ❑2 No
- I may feel not proud of my work ❑1 Yes ❑2 No

THANK YOU VERY MUCH, YOUR RESPONES ARE IMPORTANT FOR THE IMPROVEMENT OF GAVI HSS PROGRAM
